# High‐Rate and Large‐Capacity Lithium Metal Anode Enabled by Volume Conformal and Self‐Healable Composite Polymer Electrolyte

**DOI:** 10.1002/advs.201802353

**Published:** 2019-03-01

**Authors:** Shuixin Xia, Jeffrey Lopez, Chao Liang, Zhichu Zhang, Zhenan Bao, Yi Cui, Wei Liu

**Affiliations:** ^1^ School of Physical Science and Technology ShanghaiTech University Shanghai 201210 China; ^2^ Department of Chemical Engineering Stanford University Stanford CA 94305 USA; ^3^ Department of Materials Science and Engineering Stanford University Stanford CA 94305 USA; ^4^ Stanford Institute for Materials and Energy Sciences SLAC National Accelerator Laboratory Menlo Park CA 94025 USA

**Keywords:** high rates, lithium dendrites, lithium meal anodes, self‐healing polymers, volume conformal

## Abstract

The widespread implementation of lithium‐metal batteries (LMBs) with Li metal anodes of high energy density has long been prevented due to the safety concern of dendrite‐related failure. Here a solid–liquid hybrid electrolyte consisting of composite polymer electrolyte (CPE) soaked with liquid electrolyte is reported. The CPE membrane composes of self‐healing polymer and Li^+^‐conducting nanoparticles. The electrodeposited lithium metal in a uniform, smooth, and dense behavior is achieved using a hybrid electrolyte, rather than dendritic and pulverized structure for a conventional separator. The Li foil symmetric cells can deliver remarkable cycling performance at ultrahigh current density up to 20 mA cm^−2^ with an extremely low voltage hysteresis over 1500 cycles. A large areal capacity of 10 mAh cm^−2^ at 10 mA cm^−2^ could also be obtained. Furthermore, the Li|Li_4_Ti_5_O_12_ cells based on the hybrid electrolyte achieve a higher specific capacity and longer cycling life than those using conventional separators. The superior performances are mainly attributed to strong adhesion, volume conformity, and self‐healing functionality of CPE, providing a novel approach and a significant step toward cost‐effective and large‐scalable LMBs.

Lithium‐metal batteries (LMBs) can provide a major leap in energy density for the applications of electric vehicles (EVs) and smart grid, in light of growing demands for large‐scale energy storage.^1^, ^2^ As the ideal anode material, Li metal possesses a high theoretical specific capacity (3860 mAh g^−1^), the lowest electrochemical potential for Li based electrochemistry (−3.040 V vs standard hydrogen electrode) and a very low density (0.534 g cm^−3^).^3^, ^4^ However, the successful implementation of Li metal has been impeded due to the poor control of electrodeposition during the Li plating/stripping cycling, a consequence of which is the formation of mossy or dendritic lithium that can penetrate the separator, cause an internal short‐circuit, and eventually lead to battery thermal runaway. Accompanying Li dendrite growth during cycling, due to high reactivity and large surface area, the formation of solid‐electrolyte‐interphase (SEI) leads to low Coulombic efficiency (CE) and poor cycling. Moreover, unlike graphite anode that only have ≈10% volume change during lithiation/delithiation intercalation processes, Li metal is a “hostless” electrode with a virtually infinite volume change, resulting in significant internal stress accumulation, SEI collapse and high interfacial impedance upon prolonged cycling.^5^, ^6^, ^7^

Various strategies have been adopted to improve the stability of the Li metal anode and deepen our understanding, including artificial SEI layer,^8^, ^9^, ^10^ interface modification,^11^, ^12^, ^13^, ^14^, ^15^ electrolyte additives,^16^, ^17^, ^18^, ^19^ and 3D host materials.^20^, ^21^, ^22^, ^23^, ^24^ However, the performances at high current densities with high areal capacities over long cycling have not been fully explored. And, the use of porous scaffolding to deposit Li metal sacrifices energy density. A shift to the utilization of solid‐state electrolytes (SSEs) with high shear moduli that can physically suppress Li dendrite growth is a promising approach to fundamentally improve safety.^25^ Nevertheless, the performances of the current studied SSEs are far from all the requirements of high ionic conductivity (>10^−3^ S cm^−1^) at room temperature (RT), low interfacial impedance, and good electrochemical stability with electrodes. Inorganic electrolytes are too rigid to provide a good contact with electrodes, while, for solid polymer electrolytes (SPEs) with good flexibility, the ionic conductivities are much lower. One of the most effective approaches to improve the conductivity of SPEs is adding ceramic nanofillers,^26^, ^27^ reaching up to 10^−4^ S cm^−1^, which is still lower than that of liquid electrolyte. Hence, other strategies need to develop urgently.

For the cells with liquid electrolyte and commercial separator, due to the poor adhesion of the porous separator with electrodes, undesirable slippage or gap with high contact resistance could be formed at the interface between separator and Li metal anode during prolonged Li plating/stripping cycles, leading to significantly uneven Li^+^ flux distribution (**Figure** **1**a). Subsequently, Li dendrite growth and pulverization of Li metal can be expected. In contrast, a stable interface could be guaranteed by the use of a conformal electrolyte without pores (Figure 1b).

**Figure 1:**
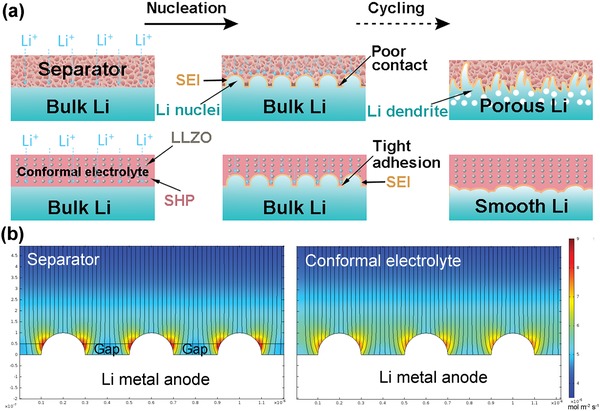
Mechanism illustration of the solid–liquid hybrid electrolyte with volume conformable functionality and strong adhesion with Li metal anode for stabilizing Li stripping/plating cycling compared with traditional liquid electrolyte‐soaked porous separator. a) Schematics illustrating the comparison of Li stripping/plating cycling with porous separator and hybrid electrolyte. b) Numerical simulations of the Li^+^ flux distribution on the surface of Li metal anode for liquid electrolyte‐soaked porous separator and hybrid electrolyte. Scale bar: 200 nm.

Herein, in our work a composite polymer electrolyte (CPE) consisting of fatty acid based self‐healing polymer (SHP)^28^, ^29^ and garnet Ga‐doped Li_7_La_3_Zr_2_O_12_ (LLZGO) nanoparticles (NPs) with volume conformal and self‐healable functionality is studied (Figure 1b). LLZGO is a good conductive Li‐ion conductor with good stability with Li metal. Previously, SHP is mostly reported as binder for Si anode,^28^, ^30^, ^31^ coating layer on Li metal anode,^29^ or as substrate polymer for electrode.^32^ However, SHP used for electrolyte is scarcely reported. Hence, the CPE membrane is expected to stabilize Li metal due to the following features: 1) strong adhesion to the Li metal with good contact; 2) volumetric accommodation with the change of electrodes to negate strain accumulation; 3) self‐healable functionality to avoid battery failure in case a crack formed during cycling; 4) uniform Li^+^ flux distribution owing to the highly conductive interfaces formed between SHP and LLZGO NPs in the CPE membrane within the cell^26^ and improved mechanical property by dispersing garnet NPs. Due to the low conductivity of the self‐healing CPE, in our work a hybrid electrolyte is adopted by adding liquid electrolyte to CPE to enhance the ionic conductivity. The Li foil symmetric cells using the hybrid electrolyte can run stably at an ultrahigh current density up to 20 mA cm^−2^ with an areal capacity of 1 mAh cm^−2^, over ultralong 1500 cycles, with the smallest polarization of 240 mV to date. The hybrid electrolyte also exhibits higher specific capacity and good cycling life for the Li/Li_4_Ti_5_O_12_ (LTO) cells, compared with commercial Celgard separator soaked with liquid electrolyte.

The importance of a conformal interface is demonstrated using numerical simulation by Comsol multiphysics. As illustrated in Figure 1b, for the traditional porous separator, gaps with higher resistance are introduced on the surface of Li metal anode in the cell geometry. It can be clearly observed that “hot spots” with increased Li^+^ flux form near the protruding nuclei, which could further amplify dendritic growth. In contrast, for the conformal electrolyte without resistive gaps, the Li^+^ flux distribution is more uniform as the reduced “hot spots.” The details on COMSOL simulation are shown in Figure S1 in the Supporting Information.

The CPE membrane was prepared via casting the solution containing SHP and LLZGO NPs in ethanol with an optimal weight ratio of 7:3. SHP is highly viscoelastic, stretchable, and sticky with good stability according to our previous results.^28^, ^30^, ^33^ showing the self‐healing functionality with a low *T*_g_ (≈−26 °C) obtained by differential scanning calorimetry. Besides, the polymer is viscoelastic at 1% strain over a frequency range of 0.01–100 Hz. And the viscoelastic property do not change significantly even in the electrolyte solutions.^29^ The CPE membrane with more amount of SHP is difficult to be prepared as a freestanding film due to the highly viscoelastic nature of the pristine SHP, while higher content of LLZGO NPs suppresses the self‐healing ability of the CPE membrane. The synthesis route of SHP is indicated in **Figure** **2**a. The X‐ray diffraction (XRD) patterns (Figure S2, Supporting Information) indicate that nanopowders with the composition of Ga_0.25_Li_6.25_La_3_Zr_2_O_12_ calcined at various temperatures could be indexed by a cubic garnet structure. The high‐resolution transmission electron microscopy (TEM) (HRTEM) image (Figure 2b) with inset selected area electron diffraction (SAED) pattern also demonstrate the cubic structure, which was quite in accordance with the XRD results. The elemental maps shown in Figure S3 in the Supporting Information reveal the uniform distribution of La, O, Zr, and Ga.

**Figure 2:**
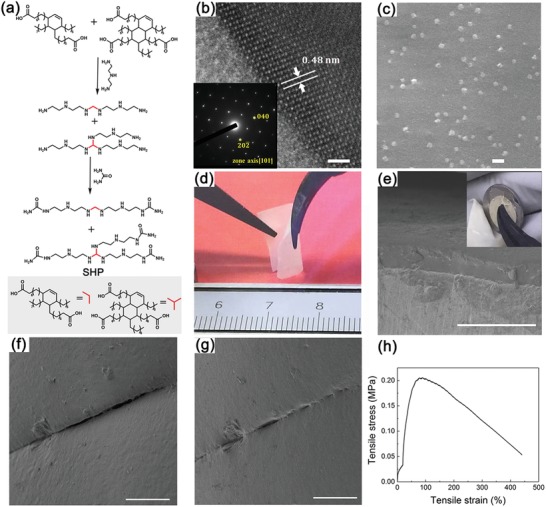
Morphology characterizations of LLZGO and CPE. a) Synthesis route of SHP. b) HRTEM image of the LLZGO NP surface (scale bar: 2 nm) and the inset is the SAED along [101] axis. c) SEM image of the CPE membrane. Scale bar: 1 µm. d) Optical images of the freestanding CPE membrane showing highly flexible feature. e) SEM images showing the CPE membrane soaked with liquid electrolyte is tightly attached onto Li foil (scale bar: 100 µm) and the inset is digital photographs showing the CPE membrane soaked with liquid electrolyte is highly sticky to Li foil (the diameter of Li foil was 1.1 cm). f,g) SEM images of the CPE surface showing the self‐healing functionality after 1 h. Scale bar 100 µm. h) Stress–strain curve of the CPE membrane.

The scanning electron microscopy (SEM) image of the CPE membrane shown in Figure 2c indicates that the LLZGO NPs are dispersed homogenously in the SHP matrix. As shown in Figure 2d, the freestanding CPE membrane is flexible with thickness of ≈25 µm. Additionally and importantly, the CPE membrane soaked with liquid electrolyte is highly sticky to Li metal foil without any visible gap from the SEM characterization as shown in Figure 2e. The digital photographs (the inset) also demonstrates that its good interfacial contact with Li metal. It can be seen in Figure S4 in the Supporting Information that the crack in the CPE membrane disappeared gradually after 2 h, demonstrating that the self‐healing and viscoelastic properties of SHP are not lost when mixed with LLZGO NPs. The self‐healing functionality of CPE was also confirmed by SEM images, showing that the crack was clearly reduced in size after 1 h (Figure 2f,g). Hence, we believe that any crack or hole in the CPE membrane formed during cycling could be self‐healed, prolonging the time of short circuit. The stress–strain curve of the CPE membrane (Figure 2h) indicates high mechanical stretchability, allowing volumetric accommodation upon Li deposition/dissolution without nonhealable damage.

The morphologies of Li foil in the Li symmetric cells using Celgard separator and hybrid electrolyte after ten cycles at a current density of 3 mA cm^−2^ with a plating/stripping capacity of 1 mAh cm^−2^ were characterized by SEM images. As shown in **Figure** **3**a, the surface of Li foil using separator indicates a typical porous needle‐like structure, with a tortuous “dead Li” layer. The dendrites with high‐surface‐area can promote SEI forming decomposition reactions, leading to the pulverization of the Li foil upon repeated plating/stripping and the quick failure of the cell. In contrast, the deposited Li on Li foil surface in contact with the hybrid electrolyte showed a smooth and dense deposition without the trace of dendritic structure (Figure 3d), ascribed to the homogeneous Li^+^ flux. And the X‐ray photoelectron spectroscopy (XPS) depth profiling of Li 1s shown in Figure S5 in the Supporting Information evidenced it is Li rather than the CPE membrane. In addition, the digital photographs shown in Figure S6 in the Supporting Information demonstrate that the Li foil surface after ten cycles maintained metallic luster as before cycling using hybrid electrolyte, while it became dark color using separator due to the accumulation of SEI. From the cross‐sectional images (Figure 3b,c,e,f), the hybrid electrolyte allowed a more compact and flat structure on Li foil surface, which is due to that the CPE membrane could tightly contact with Li foil and conform the volume change during Li plating/stripping, resulting in a stable electrolyte/Li interface during long cycling. To be noted that, with hybrid electrolyte, the contact problem of electrolyte with the electrode is also important, because a tight contact electrolyte/Li interface could reduce interfacial resistance and more effectively suppress lithium dendrite growth. In LMBs, infinite volume change of lithium metal anode would result in significant internal stress accumulation, which will result in poor interfacial contact between the polymer electrolyte and the electrode. The hybrid electrolyte composed of self‐healing CPE and liquid electrolyte is highly sticky to Li metal foil, which could guarantee the good interfacial contact contributing to the excellent battery performance.

**Figure 3:**
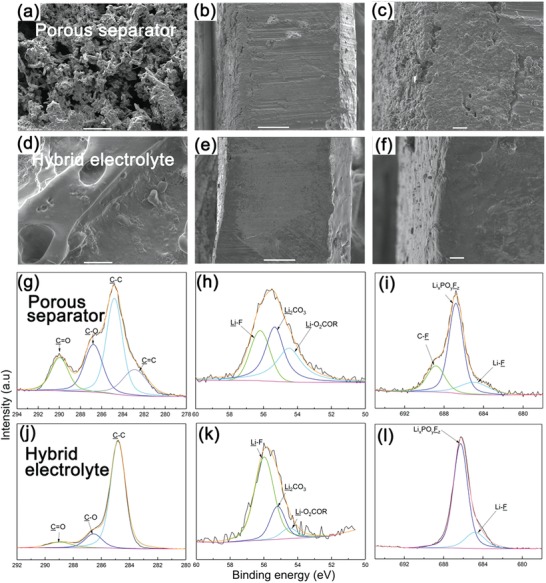
Morphology and structure characterizations of deposited Li metal. a,d) Surface and b,c,e,f) cross‐section SEM images of Li foil after ten cycles at the current density of 3 mA cm^−2^ with deposited capacity of 1 mAh cm^−2^ and the left side is the deposited lithium side. (a–c) is for porous separator and (d–f) is for hybrid electrolyte. Scale bar in (a,d): 10 µm; scale bar in (b,e): 100 µm; scale bar in (c,f): 10 µm. High‐resolution XPS of g,j) C 1s; h,k) Li 1s, and i,l) F 1s of Li foil surface after ten cycles using (g–i) porous separator and (j–l) hybrid electrolyte.

XPS experiments on Li metal foil surface in the Li symmetric cells using Celgard separator and hybrid electrolyte after ten Li plating/stripping cycles at 3 mA cm^−2^ with a capacity of 1 mAh cm^−2^ were conducted to investigate the SEI layer composition (Figure 3g–l). The C 1s spectra show three main peaks at 288.9, 286.6, and 284.8 eV corresponding to C=O, C—O, and C—C, respectively. As shown in Figure 3g, the peak of C=O shifted to 289.9 eV for the Li foil with Celgard separator due to the formation of relatively larger amount of Li_2_CO_3_,^34^ and the peak at 282.8 eV could be ascribed to the C 1s spectra of C=C. For hybrid electrolyte, the peaks of C=O, C—O, and C=C are much reduced compared with control sample, indicating more stable SEI layer with suppressed side reactions. The Li 1s spectra (Figure 3h,k) exhibit three characteristic peaks at 56.2, 55.3, and 54.5 eV ascribed to LiF, Li_2_CO_3_, and ROCO_2_Li, due to the reaction of carbonate electrolyte with Li metal. In the F 1s spectra (Figure 3i,l), the peak at 684.8 eV indicates the presence of LiF, and the two peaks at 688.6 and 686.8 eV can be designated to C–F and Li*_x_*PO*_y_*F*_z_*.^35^, ^36^ It can be seen that the relative amount of LiF is increased by the use of the hybrid electrolyte compared with the separator. It is reported that LiF could contribute to the formation of a stable and uniform SEI layer and suppressing the Li dendrite growth,^16^, ^37^, ^38^ resulting in stable Li plating/stripping cycling.

The long‐term Li plating/stripping galvanostatic cycling was conducted in the Li foil symmetric cells at the practical current densities of 3–20 mA cm^−2^ with areal capacities of 1–10 mAh cm^−2^, using hybrid electrolyte (without using battery separator) in comparison to liquid electrolyte‐soaked Celgard separator, as indicated in **Figure** **4**a–d. From the initial several cycles, the overpotential was higher at initial and reduced with continuous cycling. It can be seen from Figure 4a, for porous separator the polarization starts to gradually increase after ten cycles at 3 mA cm^−2^, due to the growth of the highly resistive SEI on Li foil surface. At higher current densities of 10 and 20 mA cm^−2^, obvious voltage fluctuations during Li deposition/dissolution processes could be observed for the cells using porous separator. It should be noted that the cells using separator exhibit obvious two peaks at the beginning and the end of each plating/stripping process, due to spatial variation in localized reaction kinetic pathways on the surface of Li metal.^39^, ^40^ In a sharp contrast, as shown in Figure 4a, the Li|Li symmetric cell based on hybrid electrolyte demonstrates flat voltage plateau with a much smaller polarization of 36 mV at the current density of 3 mA cm^−2^ over 1000 cycles (≈667 h). The hybrid electrolyte guaranteed that the Li deposits in a smooth and dense behavior without a tortuous “dead Li” layer, subsequently resulting in an extremely stable long‐term cycling. Even for the ultrahigh current densities of 10 and 20 mA cm^−2^, the hybrid electrolyte could also provide very low voltage polarization of 150 and 240 mV, respectively, and smooth voltage plateaus during Li plating/stripping cycling. The cell using hybrid electrolyte cycling at 20 mA cm^−2^ with a capacity of 1 mAh cm^−2^ indicates a stable polarization over ultralong 1500 cycles. In addition, the possibility of the hybrid electrolyte to stabilize Li metal anode with large plating areal capacity of 10 mAh cm^−2^ was also tested in the symmetric cells at high current density of 10 mA cm^−2^. The small and stable polarization over 200 h shown in Figure 4d indicates the ability of the hybrid electrolyte used in LMBs with practical high areal capacities.

**Figure 4:**
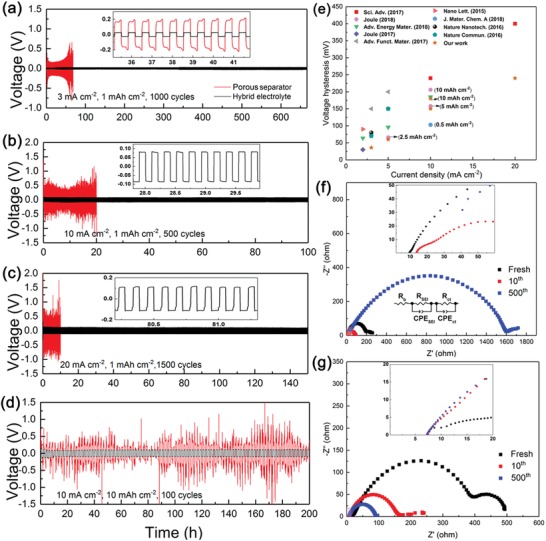
Electrical performances of the Li|Li symmetric cells. a–d) Voltage–time profiles of the symmetric cell at various high current densities and area capacities over long cycles, using hybrid electrolyte compared with porous separator. e) Voltage hysteresis of Li plating/stripping for hybrid electrolyte compared with reported data. The electrochemical impendence spectra the symmetric cells, using f) hybrid electrolyte and g) porous separator before and after cycling.

In addition, the cycling performance of the Li|Li symmetric cells using pure SHP membrane soaked with same amount of liquid electrolyte was investigated (Figure S7, Supporting Information), indicating larger potential and reduced cycling stability than the cells using hybrid electrolyte with LLZGO NPs. And, compared with the cells using pure rubber separator in reported literature, the cycling stability using hybrid electrolyte is improved.^41^ It could be expected that the addition of LLZGO NPs could result in more uniform distribution of Li‐ion flux and improved mechanical property for the CPE membrane, which could enhance the electrochemical performances of the cells.

To clearly demonstrate the ability of stabilizing Li plating/stripping, the voltage hysteresis of the symmetric cells using hybrid electrolyte after ten cycles is compared with reported data, as shown in Figure 4e. The voltage hysteresis is defined as the difference between the Li plating and stripping voltage.^11^ It can be seen that the symmetric cell using hybrid electrolyte indicates the lowest voltage hysteresis of 240 mV at the ultrahigh current density of 20 mA cm^−2^ with a capacity of 1 mAh cm^−2^, which is the lowest data to date to our knowledge. The hybrid electrolyte could guarantee a strong adhesion with Li metal and conform the volume change during repeatable Li plating/stripping, contributing to the smooth and dense structure on the surface of Li metal foil rather than a tortuous and porous surface layer, and subsequently resulting in the long‐term stable cycling.

The evolution of the electrochemical impedance spectra (EIS) upon cycling of the Li symmetric cells at 3 mA cm^−2^ with a capacity of 1 mAh cm^−2^ was carried out to support the reduced polarization and stable cycling for the hybrid electrolyte, as shown in Figure 4f,g. The equivalent circuit as inset shown in Figure 4f was used to fit the impedance spectra. The intersection with the real axis is corresponding to the sum of the electrolyte resistance and other ohmic contributions (*R*_b_) and the semicircle at high frequencies is associated with the passivation layer impedance on Li metal surface (*R*_SEI_), and the part located at low frequencies is attributed to the charge transfer resistance (*R*_ct_) and the double layer capacitance at the electrolyte/Li interface.^42^, ^43^ It can be seen that *R*_SEI_ of the cells using porous separator reduced from 207.8 to 90.4 Ω after ten cycles (Figure 4g), ascribed to the Li dendrite growth with increased active surface area, as listed in Table S1 in the Supporting Information. After long cycling for 500 cycles, the electrolyte resistance and the interfacial resistance greatly increase to 19.0 and 1612.9 Ω, suggesting the excessive consumption of liquid electrolyte, the accumulation of thick SEI layer and the pulverization of Li metal foil. While, for the cells based on hybrid electrolyte, although a larger interfacial resistance could be observed before cycling, it reduced after ten cycles and even reduced to as small as 92.1 Ω after 500 cycles (Figure 4f), which could be ascribed to the improved contact of Li foil with the hybrid electrolyte after cycling. Moreover, due to the highly stick of hybrid electrolyte, Li foil could hardly react with carbonate electrolyte to form thick and unstable SEI layer. And the hybrid electrolyte indicates electrolyte resistance of 6.9 Ω after 500 cycles, which is much smaller than the liquid electrolyte. Hence, from the EIS results, it revealed that the symmetric cells using the hybrid electrolyte showed stable and small electrolyte resistance and interfacial resistance over ultralong Li plating/stripping cycles, which results in the low polarization with cycling. Besides, It can been calculated that the total conductivity of the CPE membrane is about 10^−10^ S cm^−1^ at room temperature according to the impedance spectra of CPE membrane shown in Figure S8 in the Supporting Information. And, the total conductivity of hybrid electrolyte (the CPE membrane with liquid electrolyte) is about 10^−3^ S cm^−1^ at room temperature, which is much higher than the CPE membrane. Therefore, the electronic conductivity of the CPE membrane could be neglected compared with the liquid electrolyte. Thus we believe it is not the electronic conductivity dominating during Li plating/stripping.

Moreover, the cells using Li_4_Ti_5_O_12_ (LTO) as the working electrode coupled with Li metal foil have been tested. A thin layer of polyvinylidene fluoride (PVDF) with thickness of ≈5 µm was coated on the CPE membrane to prevent the decomposition of SHP at the voltage higher than 1 V when contacting with LTO.^29^ The voltage profiles as a function of time for the Li|LTO cells using porous separator and hybrid electrolyte/PVDF at 0.2C and 1.0C are shown in **Figure** **5**a,b. The cells based on hybrid electrolyte could deliver a higher reversible capacity of 157 and 149 mAh g^−1^ at 0.2C and 1.0C, in comparison to the cells using commercial separator with specific capacity of 143 and 129 mAh g^−1^. And the cells with the hybrid electrolyte/PVDF showed a high specific capacity of 129 mAh g^−1^ at 2C (Figure S9, Supporting Information). To confirm the contribution from the hybrid electrolyte rather than the PVDF layer, the Li|LTO cell using a single membrane of PVDF (thickness ≈25 µm) soaked with same amount of liquid electrolyte was studied and showed poor electrochemical performances than that based on hybrid electrolyte (Figure S10, Supporting Information). The Li foil symmetric cell with pure PVDF membrane was also tested at a current density of 3 mA cm^−2^ with a capacity of 1 mAh cm^−2^. However, the cell shorted after about 80 cycles (Figure S11, Supporting Information). Thus we believed that with only the PVDF membrane couldn't inhibit the growth of lithium dendrite. Moreover, as shown in Figure 5c, the cells based on hybrid electrolyte demonstrated a better rate capability. The Li|LTO cells with hybrid electrolyte also exhibited good cycling performance with a high capacity retention of 99.4% for 120 cycles at 0.2C, compared with the separator of 95.4% (Figure 5d). The good electrochemical performance for the cells based on hybrid electrolyte is attributed to the uniform reaction kinetic pathways at electrolyte/Li interface.

**Figure 5:**
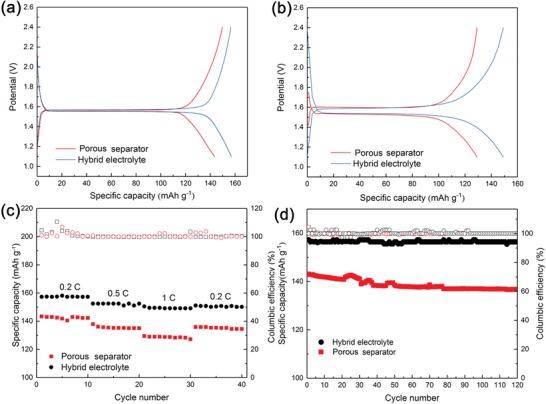
Electrochemical performances of the Li|LTO cells. Discharge/charge voltage profiles of the Li|LTO cells using porous separator and hybrid electrolyte/PVDF at the rate of a) 0.2C and b) 1.0C. c) Rate capability of the Li|LTO cells based on porous separator and hybrid electrolyte/PVDF. d) Cycling performance and CE of the Li|LTO cells using porous separator and hybrid electrolyte/PVDF.

In summary, we have demonstrated a CPE membrane consisted of SHP and LLZGO NPs, with strong adhesion, volume conformity and seal‐healing functionality for stabilizing Li metal anode. A uniform, smooth, and dense structure of deposited Li metal without dendritic growth and pulverization could be observed after repeatable cycling by the use of hybrid electrolyte composed of CPE soaked with liquid electrolyte. The Li foil symmetric cells based on hybrid electrolyte can deliver stable cycling performances at current density range of 3–20 mA cm^−2^ with a deposited capacity of 1 mAh cm^−2^ for 500–1500 cycles with remarkably low potentials. The voltage hysteresis of 240 mV at the ultrahigh current density of 20 mA cm^−2^ was achieved, which is the lowest value to date to our knowledge. The Li/LTO half‐cells based on the hybrid electrolyte could achieve a stable cycling after 120 cycles for at 0.2C with a high specific capacity of 157 mAh g^−1^ and a capacity retention of 99.4%. This simple design on hybrid electrolyte indicates the feasibility of scale‐up toward next‐generation LMBs with high energy density, long cycling life and good safety.

## Experimental Section

*Fabrication of LLZGO Nanoparticles*: Ga_0.25_Li_6.25_La_3_Zr_2_O_12_ (LLZGO) was prepared via the Pechini sol–gel method.^44^ And Ga was doped to contribute to the formation of cubic phase. LiNO_3_ (>99.99%, 0.0385 mol), La(NO_3_)_3_ (>99.99%, 0.0168 mol), Ga(NO_3_)_3_ (>99.99%, 0.0014 mol), and Zr(C_5_H_7_O_2_)_4_ (>99.99%, 0.0112 mol) was first dissolved in ethanol/H_2_O solutions and kept *V*_ethanol_:$V_{H_{2}O}$ = 4:1. LiNO_3_ was more added 10% to compensate the Li element loss during the high temperature treatment. And then citric acid was added and milk like sol was obtained. The sol was first heated at 60 °C for 4 h and then heated to 250 °C to dry the sample completely. A brown color porous gel could be obtained in the end. Then the brown samples were calcined at 800 °C for 5 h in air and cubic LLZGO could be obtained.

*Fabrication of Composite Polymer Membrane*: SHP was synthesized according to refs. 29, 30, 33. First, certain amount of SHP was dissolved in ethanol to get a homogeneous solution. And then LLZGO nanopowder was added to the solutions and stirred overnight to get a homogenous slurry. The slurry was then cast on a glass plate with a doctor blade. Finally, the membrane was dried in high vacuum overnight to completely remove the trace of solvent. The hybrid electrolyte consisted of the CPE membrane and liquid carbonate electrolyte (60 µL) (lithium hexafluorophosphate (LiPF_6_, 1 m) in ethylene carbonate and diethyl carbonate (EC/DEC, 1:1 by volume).

*Characterizations and Electrochemical Measurement*: XRD (Bruker D8 Advance) was used for phase identification using Cu Kα radiation of 0.15406 nm. The morphologies of samples were examined by SEM (JEOL 7800). TEM characterizations were carried out with a JEM 2100 plus thermionic transmission electron microscope operated at 200 kV. The elemental maps were acquired with energy dispersive spectroscopy detector Oxford IT100. XPS spectra were recorded with the ThermoFisher ESCA 250XI using an Al Kα (λ = 0.83 nm, *hυ* = 1486.7 eV). And the X‐ray source was operated at 2 kV, 20 mA. The C 1s neutral peak at 284.8 eV was used as the reference to correct for the shift caused by surface‐charging effects. CR‐2032‐type coin cells were assembled in argon‐filled glove box with Celgard 2325 as the separator and 60 µL liquid electrolyte of 1 m LiPF_6_ in ethylene carbonate (EC) and diethyl carbonate (DEC) (1:1 by volume). The electrode slurry was prepared by blending the LTO powder (MTI Corporation), super P (MTI Corporation), and PVDF binder (MTI Corporation) with a weight ratio of 8:1:1 in the *N*‐methyl‐2‐pyrrolidone (NMP) (Sigma‐Aldrich) solvent. And then the slurry was casted on Cu foil with a doctor‐blade. PVDF membrane was prepared by resolving the PVDF powder in NMP solvent and cast onto a glass vessel. All the obtained samples were dried overnight in a vacuum oven at 60 °C to remove the solvent completely. For the LTO/Li half cells assembly, the PVDF membrane (≈5 µm) was coated on the CPE membrane. The electrical conductivity investigated using a.c.‐impedance spectroscopies were recorded by a Biologic VSP potentiostat over the frequency range of 0.10 Hz to 7 MHz. The electrochemical performances of batteries were tested via LAND batteries test systems.

## Conflict of Interest

The authors declare no conflict of interest.

## Supporting information

Supplementary
