# Supplementary material for: High‐Rate and Large‐Capacity Lithium Metal Anode Enabled by Volume Conformal and Self‐Healable Composite Polymer Electrolyte
Source: Adv Sci (Weinh). 2019 Mar 1;6(9):1802353. doi: 10.1002/advs.201802353 (PMC6498105; doi:10.1002/advs.201802353)
Supplement: Supplementary file 1 — Supplementary [file ADVS-6-1802353-s001.pdf]

Copyright WILEY-VCH Verlag GmbH & Co. KGaA, 69469 Weinheim, Germany, 2019.

ADVANCED SCIENCE
Open Access

Supporting Information

for *Adv. Sci.,* DOI: 10.1002/advs.201802353

High-Rate and Large-Capacity Lithium Metal Anode Enabled by Volume Conformal and Self-Healable Composite Polymer Electrolyte

*Shuixin Xia, Jeffrey Lopez, Chao Liang, Zhichu Zhang, Zhenan Bao, Yi Cui, and Wei Liu**

Copyright WILEY-VCH Verlag GmbH & Co. KGaA, 69469 Weinheim, Germany, 2019.

Supporting Information

High-rate and large-capacity lithium metal anode enabled by volume conformal and self-healable composite polymer electrolyte

*Shuixin Xia, Jeffrey Lopez, Chao Liang, Zhichu Zhang, Zhenan Bao, Yi Cui, Wei Liu**

Dr. S. Xia, C. Liang, Z. Zhang, Prof. W. Liu
School of Physical Science and Technology
ShanghaiTech University
Shanghai 201210, China
Email: liuwei1@shanghaitech.edu.cn

Dr. J. Lopez, Prof. Z. Bao
Department of Chemical Engineering
Stanford University
Stanford, California 94305, USA

Prof. Y. Cui
Department of Materials Science and Engineering
Stanford University
Stanford, California 94305, USA
Stanford Institute for Materials and Energy Sciences
SLAC National Accelerator Laboratory
Menlo Park, California 94025, USA

Contents

1. Supporting figures

2. Supporting tables

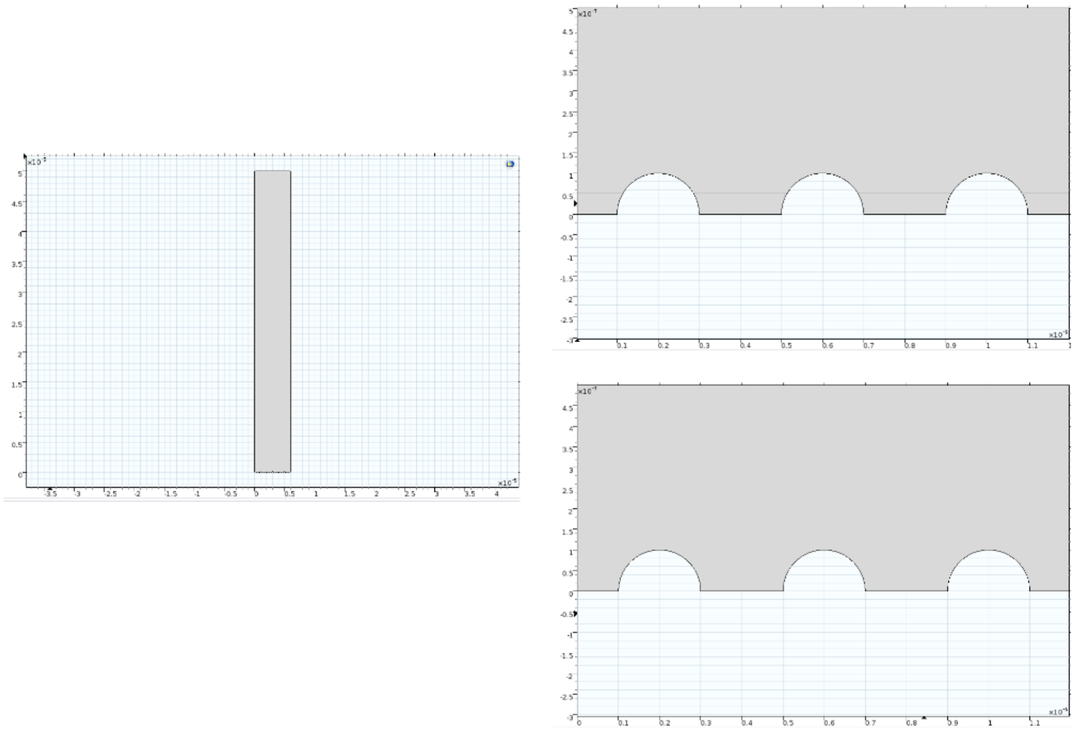

Figure S1. The cell geometries in COMSOL simulation using traditional liquid electrolyte-soaked porous separator and hybrid conformal electrolyte. The distance between two electrodes is 50 μm. The diameter of Li nuclei is 200 nm. For porous separator, gaps with high resistance are formed on the surface of Li metal anode. The overpotential of deposition was set to -200 mV vs. Li/Li^+ at the working electrode. The diffusion coefficient of Li^+ was set to 1×10^{-5} cm^2 s^{-1}. The diffusion coefficient of Li^+ of the gaps is one order of magnitude lower than that of the separator soaked with electrolyte. The electrodeposition module in COMSOL uses the Einstein relation ($D = \mu k_b T/q$).

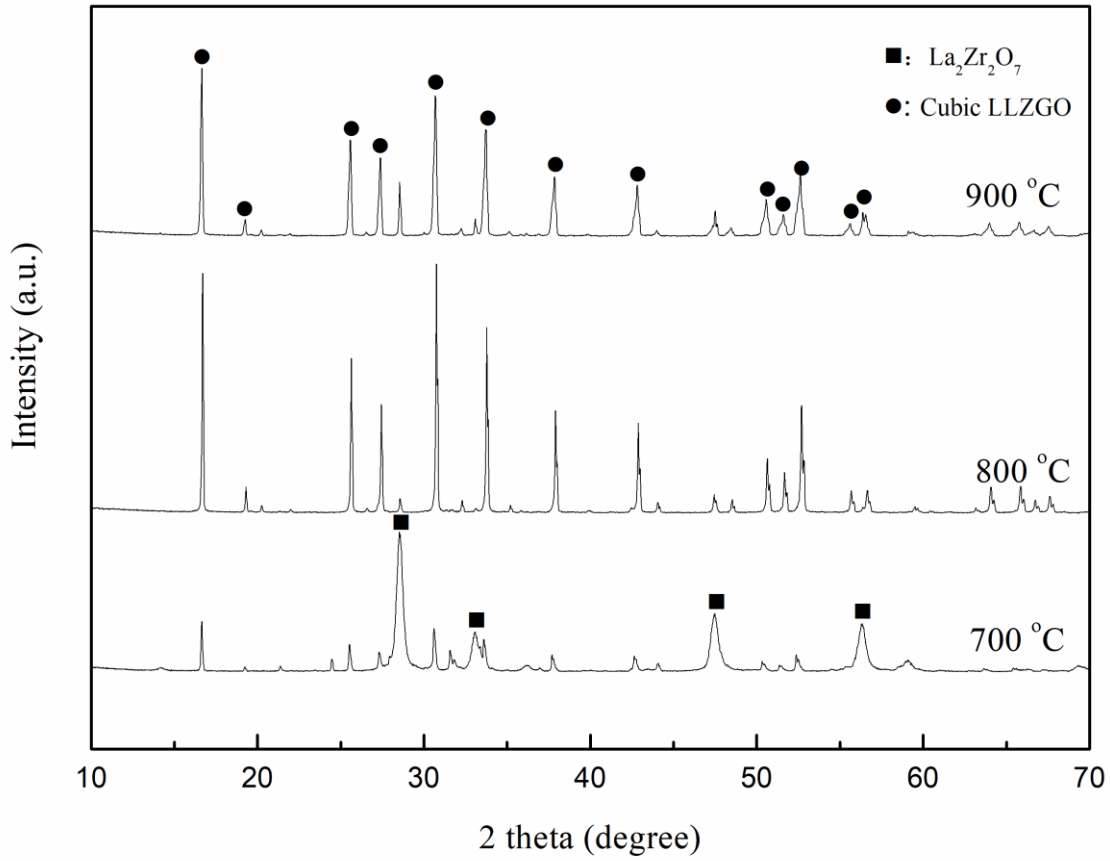

Figure S2. XRD patterns of the LLZGO nanopowders with the composition of $Ga_{0.25}Li_{6.25}La_3Zr_2O_{12}$ calcined at various temperatures.

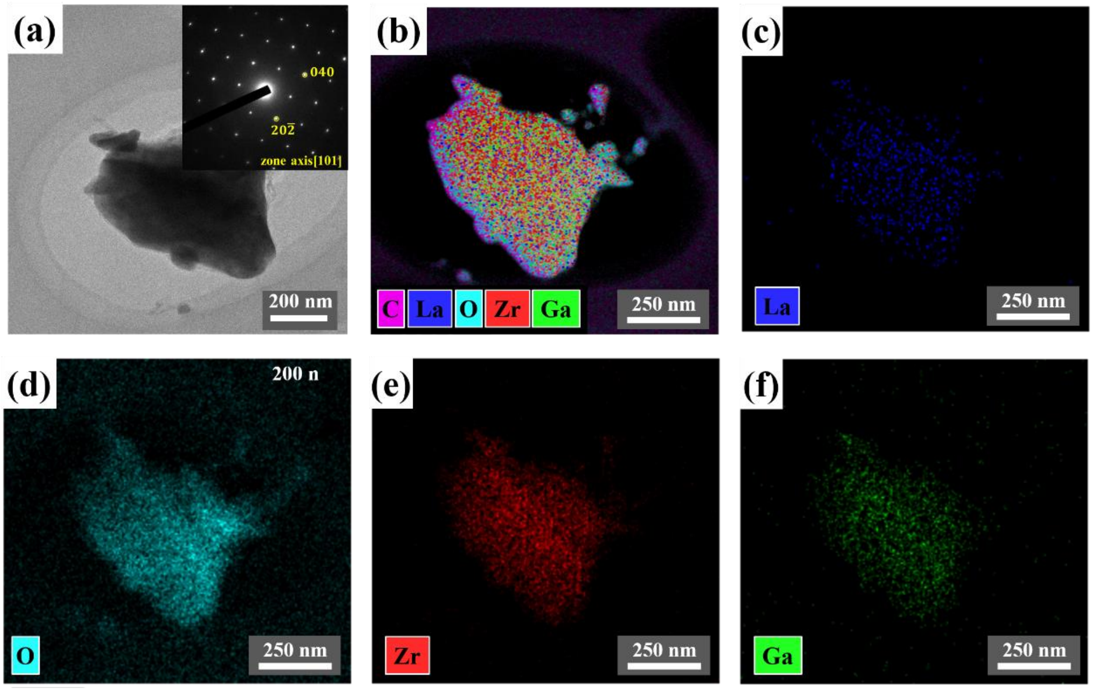

Figure S3. a) Low magnification TEM image of a LLZGO NP. b-f) The elemental maps of La, O, Zr, Ga, respectively.

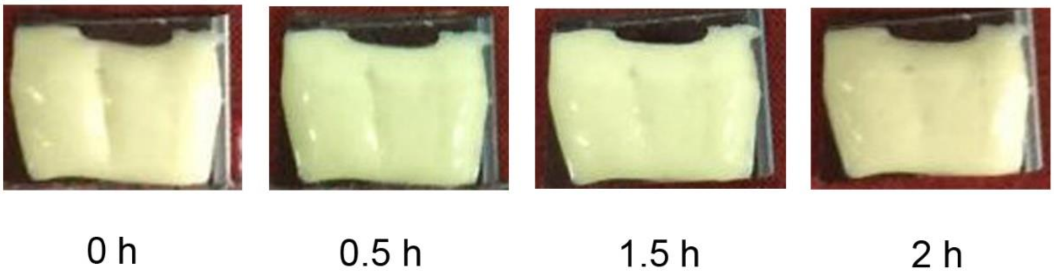

Figure S4. Digital photographs of the CPE membrane showing self-healing functionality. (the length of the Si substrate is 0.6 cm)

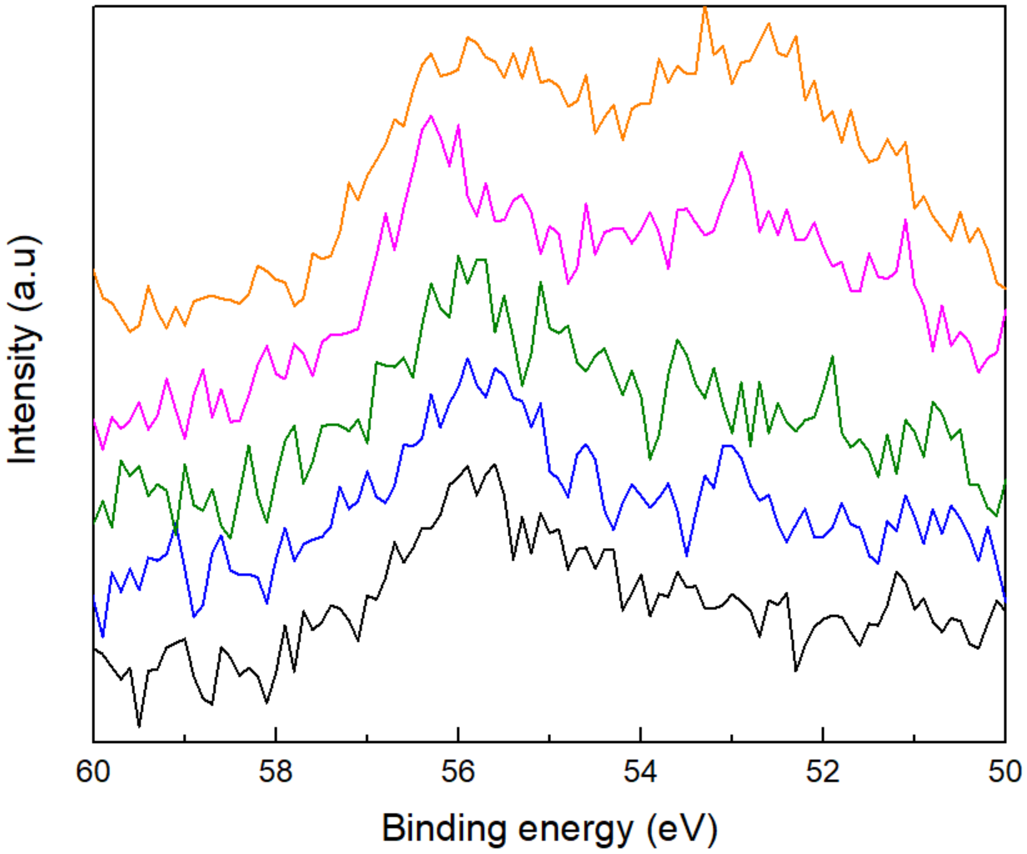

Figure S5. XPS depth profiling of Li 1s of Li metal from Li|Li symmetric cells with hybrid electrolyte after 10 cycles at the current density of 3 mA cm^{-2} with deposited capacity of 1 mAh cm^{-2}. Curves from bottom to top represent the spectra acquired after sputtering for 0, 10, 30, 60, 90 min.

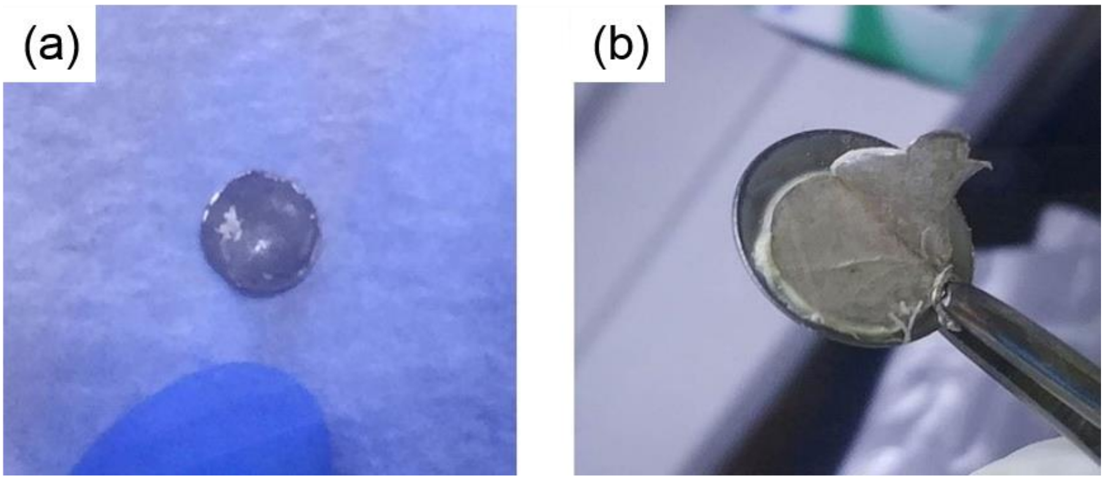

Figure S6. Digital photographs of the Li foil surface after 10 cycles by the use of hybrid electrolyte b) compared with liquid electrolyte a) at the current densities of 3 mA cm^{-2} with plating capacities of 1 mAh cm^{-2}.

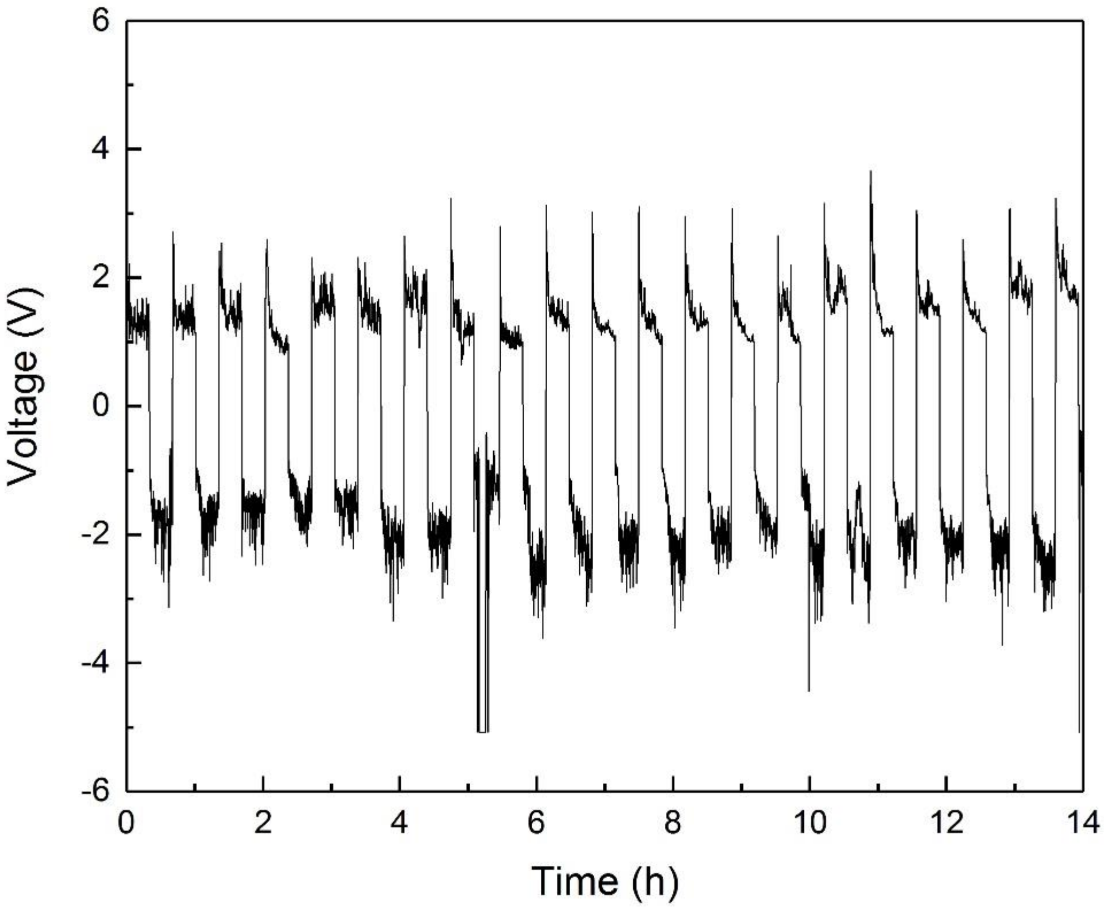

Figure S7. Voltage-time profile of the Li|Li symmetric cells using pure SHP membrane soaked with liquid electrolyte at the current densities of 3 mA cm^{-2} with capacities of 1 mAh cm^{-2}.

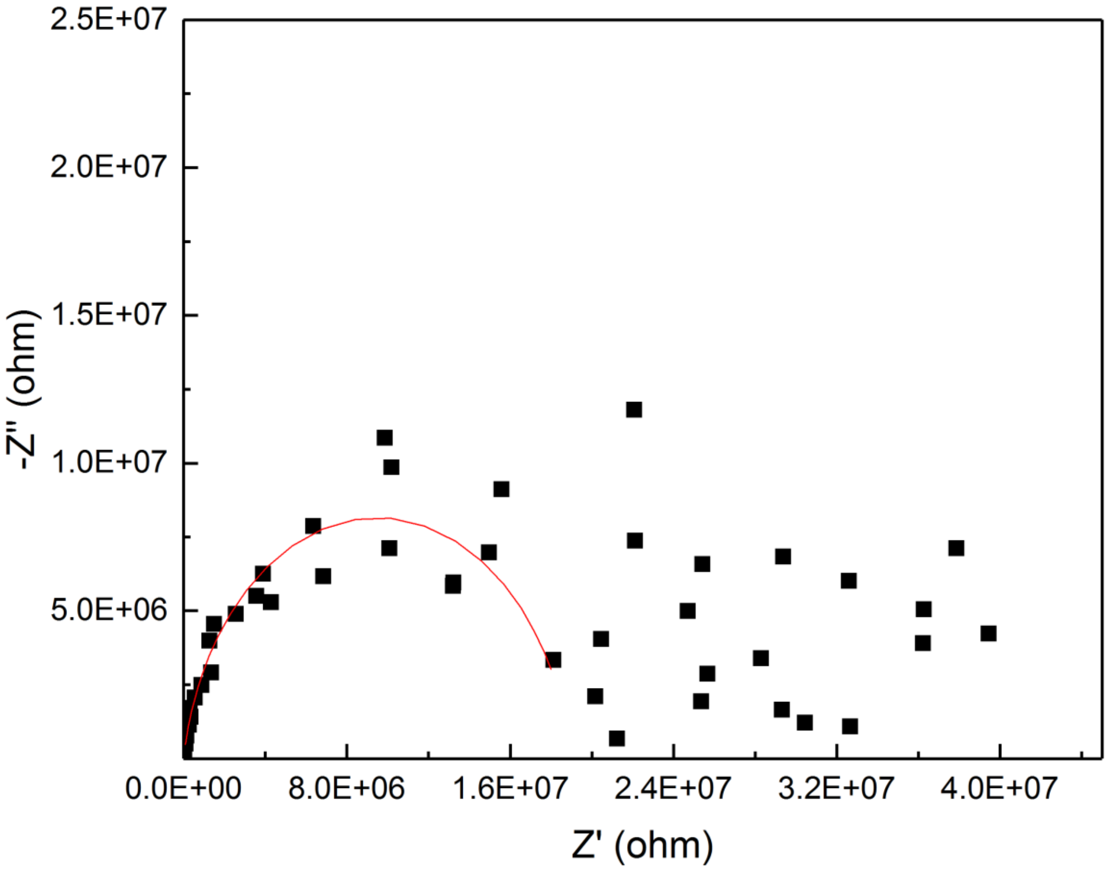

Figure S8. Experimental and fitting impedance spectra for the CPE membrane tested at 30 ^{o}C.

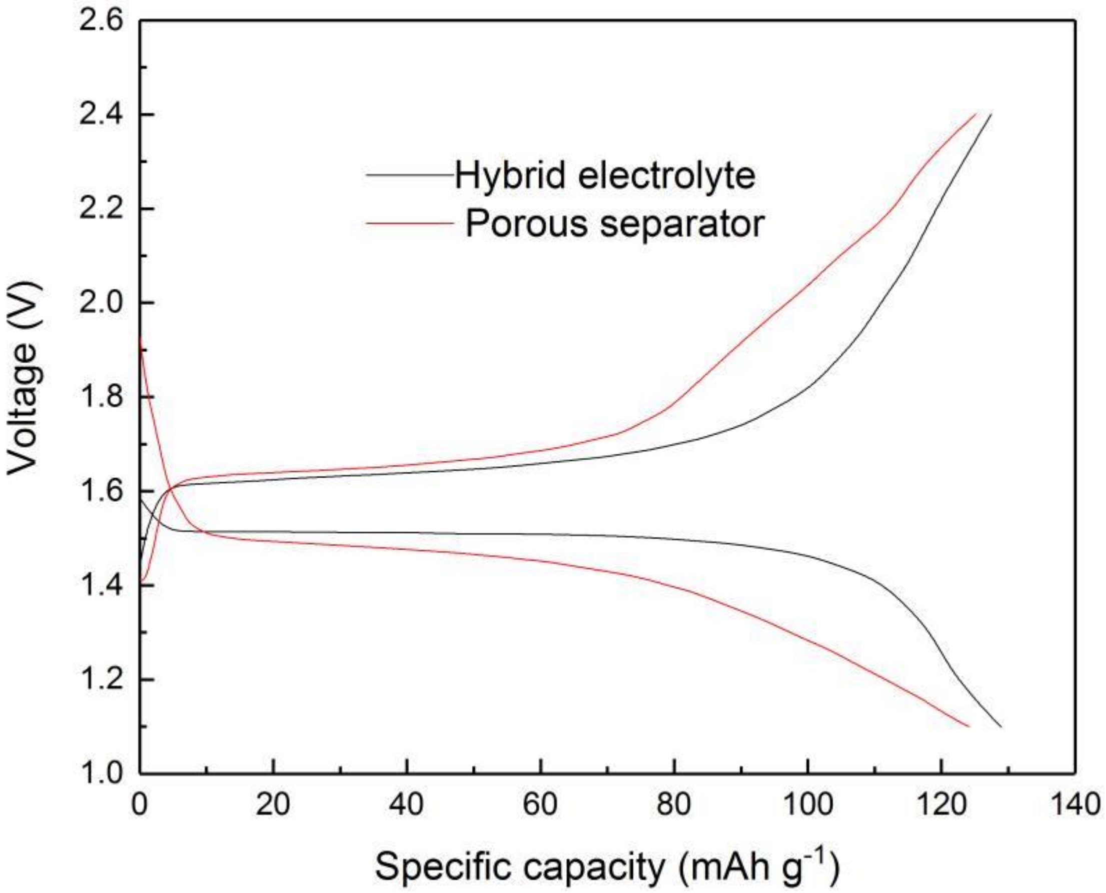

Figure S9. Discharge/charge voltage profiles of the Li|LTO cells using porous separator and hybrid electrolyte/PVDF at 2 C.

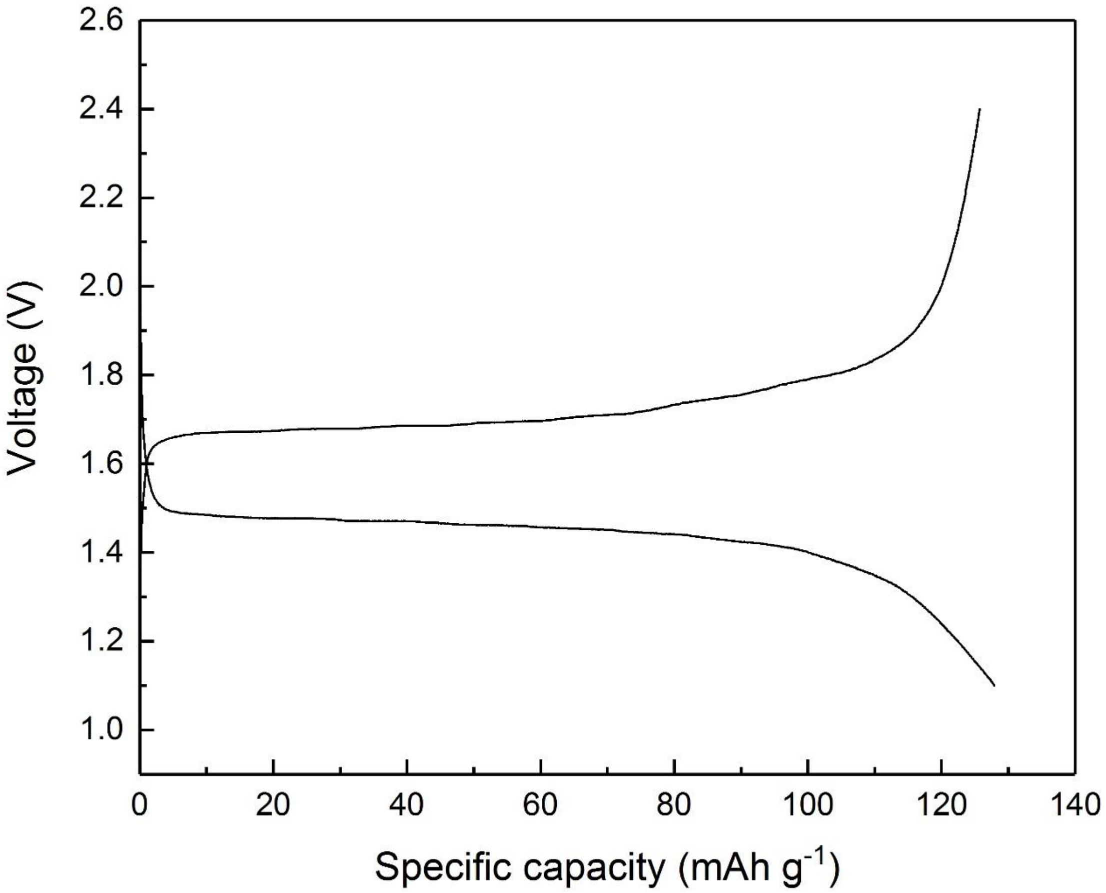

Figure S10. Discharge/charge voltage profiles of the Li|LTO cell using a single membrane of PVDF soaked with liquid electrolyte at 0.2 C.

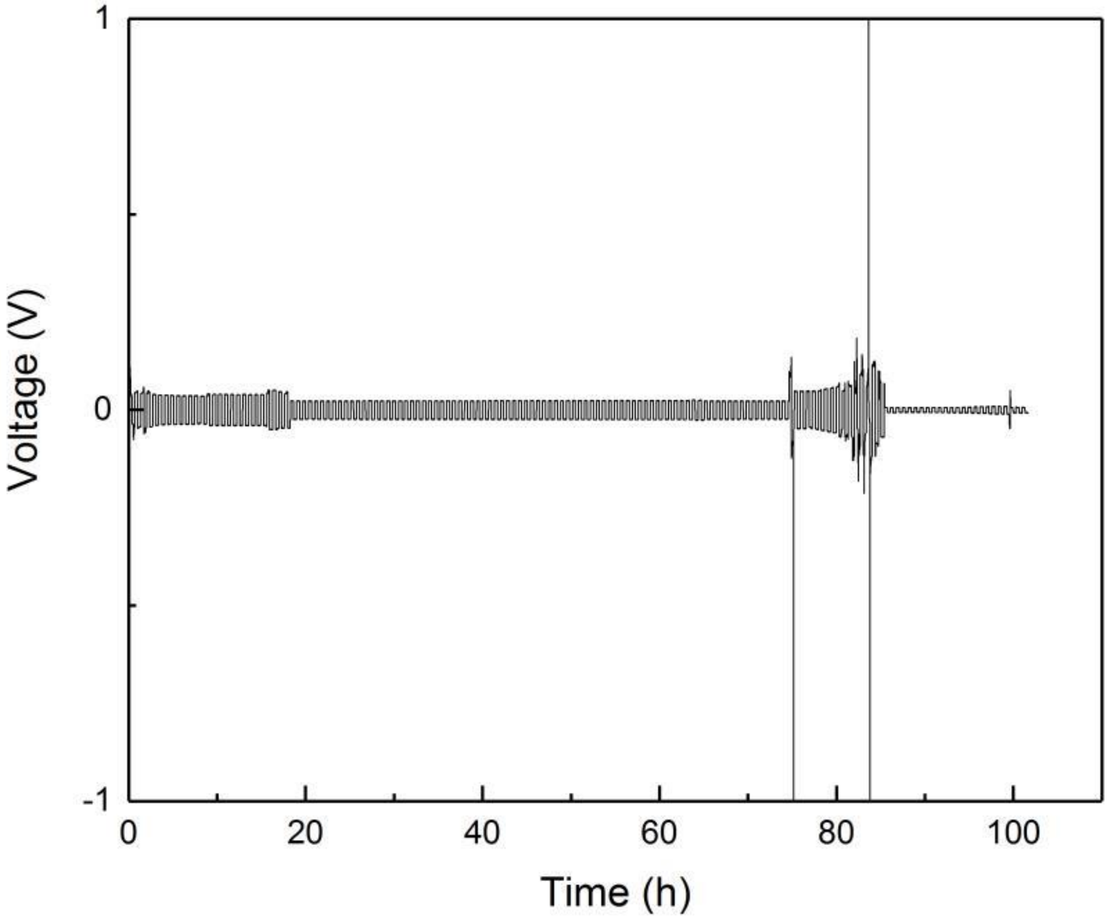

Figure S11. Electrical performances of the Li|Li symmetric cells with pure PVDF membrane soaked with liquid electrolyte at the current densities of 3 mA cm^{-2} with a capacity of 1 mAh cm^{-2}.

Table S1. Electrolyte and interfacial resistances for the Li symmetric cells at 3 mA cm^{-2} with a capacity of 1 mAh cm^{-2} before and after various cycles.

Sample	Cycle number	R_b (ohm)	R_{SEI} (ohm)
Celgard separator	0	9.6	207.8
	10	13.6	90.4
	500	19.0	1612.9
Hybrid electrolyte	0	8.1	419.2
	10	7.2	162.9
	500	6.9	92.1
